# Distinct Clinical Presentations and Outcomes of Hospitalized Adults with the SARS-CoV-2 Infection Occurring during the Omicron Variant Surge

**DOI:** 10.3390/healthcare11121703

**Published:** 2023-06-10

**Authors:** Jianli Niu, Myeongji Kim, Ayesha T. Jalal, Jessica E. Goldberg, Elsa M. Acevedo Martinez, Nathalie P. Suarez Moscoso, Heysu Rubio-Gomez, Daniel Mayer, Alvaro Visbal, Candice Sareli, Paula A. Eckardt, Aharon E. Sareli

**Affiliations:** 1 Office of Human Research, Memorial Healthcare System, Hollywood, FL 33021, USA; 2 Department of Internal Medicine, Memorial Healthcare System, Hollywood, FL 33021, USA; 3 Division of Infectious Disease, Memorial Healthcare System, Hollywood, FL 33021, USA; 4 Division of Critical Care Medicine, Memorial Healthcare System, Hollywood, FL 33021, USA

**Keywords:** COVID-19, Omicron variant, hospitalizations, incidental COVID-19, in-hospital mortality, intensive care services

## Abstract

The COVID-19 Omicron variant has imposed a tremendous burden on healthcare services. We characterized the types of the Omicron variant-associated hospitalizations and their associations with clinical outcomes. Consecutive adults hospitalized with COVID-19 during the Omicron variant surge period of 1–14 January 2022, were classified into one of three groups based on their clinical presentations on admission: Group 1—primary COVID-19; Group 2—extrapulmonary manifestations of COVID-19; and Group 3—incidental COVID-19. Of the 500 patients who were hospitalized, 51.4% fell into Group 1, 16.4% into Group 2, and 32.2% into Group 3. The patients in Groups 1 and 2 were older, with higher proportions of comorbidities than patients in Group 3. The Group 1 patients had the highest mortality rate (15.6%), followed by Group 2 (8.5%), and Group 3 (0.6%), with adjusted odds ratios (OR) of 22.65 (95% confidence interval [CI], 2.75–239.46; *p* = 0.004) and 10.95 (95% CI, 1.02–117.28; *p* = 0.048), respectively, compared to Group 3. Those in Group 1 showed a greater utilization of intensive care services (15.9%), followed by Group 2 (10.9%), and Group 3 (2.5%), with adjusted ORs of 7.95 (95% CI, 2.52–25.08; *p* < 0.001) and 5.07 (95% CI, 1.34–19.15; *p* = 0.017), respectively, compared to Group 3. The patients in Groups 1 and 2 had longer hospitalization stays than the patients in Group 3 (*p* < 0.001 and *p* = 0.002, respectively). Older age (≥65 years) was an independent factor associated with longer hospital stays (OR = 1.72, 95% CI, 1.07–2.77). These findings can help hospitals prioritize patient care and service planning for future SARS-CoV-2 variants.

## 1. Introduction

The first case of the SARS-CoV-2 Omicron variant (B.1.1.529) in the United States was reported on 1 December 2021 [1]. By late December 2021, it became the predominant strain, and by 15 January 2022, it represented 99.5% of sequenced specimens in the US [1], resulting in surges in COVID-19 cases and associated hospitalizations [1,2]. Although preliminary evidence exists regarding this variant’s transmissibility, increases in SARS-CoV-2 Omicron infections have not been associated with parallel increases in the severity of illness. Compared to the Delta variant, hospitalized patients during the Omicron wave had less severe illness and shorter hospital stays [3,4,5]. This observation has partly been attributable to humoral immunity acquired through vaccination and/or natural infection [2,6,7,8]. However, several studies have reported reduced neutralization of the Omicron variant in individuals with complete (two- or three-dose) mRNA vaccine series [9], and from patients with prior SARS-CoV-2 infection [10]. Some public health experts have argued that the current classification systems do not fully capture the complexity of the Omicron variant infections and hospitalizations. The reasoning is that not all hospitalized patients are admitted solely due to COVID-19, and that some patients may be hospitalized for an unrelated reason while incidentally being diagnosed with COVID-19, or are hospitalized due to a COVID-19-induced exacerbation of underlying conditions [11,12,13,14]. This raises a particular concern regarding the severity of illness caused by the Omicron variant, as the number of individuals hospitalized due to COVID-19 is a key indicator of the severity of the virus. The accurate reporting and classification of hospitalizations are crucial for assessing the severity and clinical outcomes associated with the SARS-CoV-2 Omicron variant [11,13,15,16,17].

Memorial Healthcare System, a public health care system consisting of four adult acute-care hospitals across South Florida, serves a diverse community that has the greatest proportion of an older population in the United States. The first known case of the SARS-CoV-2 Omicron variant infection in Florida was reported on 7 December 2021 [18]. Since mid-December, Florida has experienced an extremely rapid spread of the Omicron variant, with a hospitalization rate of 89.2 per 100,000 population. To investigate how patients hospitalized with COVID-19 during the SARS-CoV-2 Omicron wave experienced their illness in this population, we conducted a retrospective cohort study of consecutive adult patients who were hospitalized at Memorial Healthcare System with SARS-CoV-2 infection in a two-week study interval, 1–14 January 2022. We classified the causes of these COVID-19 hospitalizations, and compared risk of severe clinical outcomes among these patients based on their distinct clinical presentations, as well as the epidemiologic characteristics of these patients, including their history of vaccination and age, in the hope of better understanding the severity of illness and health care utilization associated with the pandemic of the SARS-CoV-2 Omicron variant.

## 2. Materials and Methods

### 2.1. Study Design and Setting

This was a retrospective observational study conducted at Memorial Healthcare System, Hollywood, Florida. According to the information on SARS-CoV-2 circulation in the US, the surge of the Omicron variant began in mid-December 2021 [1,2]. As such, consecutive adult patients (≥18 years) who were hospitalized in Memorial Healthcare System with confirmed SARS-CoV-2 infections between 1–14 January 2022 were included in the study. Patients without a positive SARS-CoV-2 test were excluded to ensure that the cases included in the study were confirmed cases of COVID-19, as determined by laboratory testing for the qualitative detection of the SARS-CoV-2 nucleic acid in a nasopharyngeal swab sample via a real-time polymerase chain reaction using Roche cobas^®^ SARS-CoV-2 assay on the cobas^®^ 6800 system (Roche Molecular Systems, Branchburg, NJ, USA). The study was approved by the Institute Review Board of Memorial Healthcare System (MHS.2022.015). Informed consent was waived, as this was a secondary data analysis of existing data.

### 2.2. Data Collection

Patients were identified using the electronic health record (EHR) by extracting all patients with a positive SARS-CoV-2 test during the study period. The data were manually abstracted from EHR files utilizing a standardized data collection instrument. The patients’ baseline characteristics, including age, gender, race and ethnicity, and underlying conditions such as obesity (defined as a calculated body mass index ≥ 30 kg/m^2^), smoking history, comorbidities, active malignancy, history of malignancy, history of transplantation (solid or bone marrow), history of previous infection, vaccination status, admission symptoms, need for oxygen supply and mechanical ventilation, admission to intensive care unit (ICU), COVID-19–directed treatments, complications, hospital length of stay (LOS), ICU LOS, and in-hospital deaths, were obtained. Laboratory data were also collected within 24 h of hospitalization, including neutrophil and lymphocyte counts, serum levels of C-reactive protein (CRP), D-dimer, and lactate dehydrogenase (LDH).

For patients who were vaccinated, the vaccine type, the date of vaccination, and number of doses were collected based on electronic health records linking to credible vaccination registries. Patients were considered fully vaccinated if they received the second dose of vaccine as per the vaccination protocol, at least 14 days before symptom onset [19]. If patients received an additional dose of a vaccine, this was considered a booster dose. Individuals who had no record of vaccination against COVID-19 or were vaccinated with a single dose or <14 days after receipt of the second dose before illness onset were defined as unvaccinated patients [19].

The study included each patient only once in the analysis. In the cases of hospital readmissions during the study period, only the first hospital admission was included in the analysis.

### 2.3. Outcome Measures and Definitions

The cases of hospitalized patients who had a positive SARS-CoV-2 test result upon clinical admission or during their stay in hospital were classified into one of the following three categories: Group1—Primary COVID-19, patients who were admitted to the hospital due to only COVID 19 pneumonia or respiratory infection; Group 2—Extrapulmonary manifestations of COVID-19; Group 3—Incidental COVID-19, patients who were admitted to the hospital for a diagnosis other than COVID-19 but were incidentally diagnosed with COVID-19 on the routine testing of all admitted patients. The classifications were performed by study investigators through evaluation of the patient’s clinical presentations at admission. A classification table (Table 1) was developed and used to classify the patients based on a review of the patients’ EHR files.

The outcomes of interest in the study included the number and proportion of patients falling into one of the three groups, the need for intensive care, in-hospital mortality, hospital LOS, and ICU LOS among the three groups of patients. An in-hospital death was defined as an expired discharge status in a hospitalized patient who tested positive for COVID-19. Hospital LOS was defined as the number of days from admission until first discharge from the hospital. All of the patients in the study were followed from admission to discharge/death from the hospital. Stays that were censored by death were excluded, as they did not represent a complete hospital stay. Additionally, clinical and laboratory characteristics across the three groups were compared.

### 2.4. Statistical Analysis

Descriptive statistics were performed to summarize the demographic, clinical and laboratory characteristics, and outcomes of the patients. The Shapiro–Wilk test was used to assess the normality of continuous variables. The mean and standard deviation (SD) or, if variables were not normally distributed, the median and interquartile range (IQR) were calculated for continuous variables, and frequencies and percentages were calculated for the categorical variables. *t*-tests or Mann–Whitney U tests were used to evaluate continuous variables, and Fisher’s exact tests or chi-squared tests were performed to compare categorical variables, as appropriate. The Kruskal–Wallis test followed by Dunn’s test was used to compare differences in lab findings and hospital/ICU LOSs among the three groups. Logistic regression analyses were performed to estimate odds ratios (ORs) with 95% confidence intervals (CIs) of ICU admission and in-hospital mortality among the three groups. Then, the models were adjusted for confounders to calculate adjusted ORs with 95% CIs. Subgroup analyses were conducted based on age and vaccination status. For the age subgroup analysis, patients were categorized according to being younger than 65, and being 65 years or older. The risks of ICU admission and in-hospital death stratified by vaccination status and age (<65 and ≥65 years) were examined using the same methodology as the that used in the main analysis. Patient-level factors that were associated with longer patient LOSs (defined as a LOS > 3 days) were examined using univariate and multivariate logistic regression models. Variables that were significantly associated with a LOS > 3 days with a significance level of *p* < 0.25 were selected for possible inclusion in multivariate logistic regression models to predict patient LOSs > 3 days. A two-tailed, *p*-value of <0.05 was considered statistically significant. The analyses were performed using statistical packages SPSS V.28.0 (IBM Corp) and GraphPad Prism V.9.0 (San Diego, CA, USA).

## 3. Results

**Figure 1:**
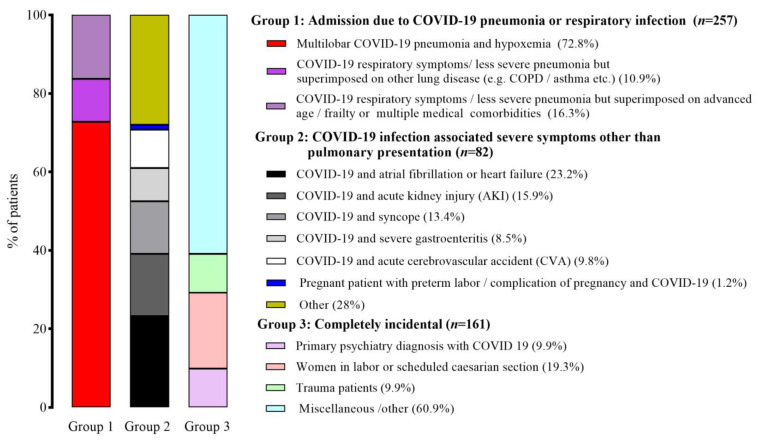
Distribution of hospitalizations according to the standardized classification criteria for COVID-19-associated hospital admission.

A total of 500 patients were hospitalized with Omicron variant infections during 1–14 January 2022. Figure 1 shows the distribution of hospitalizations according to the classification criteria used on admission. A total of 257 (51.4%) patients fell into Group 1, 82 (16.4%) into Group 2, and 161 (32.2%) into Group 3.

Table 2 shows patient characteristics stratified according to group. The population analyzed had median ages of 73 (IQR, 61–82), 74 (IQR, 62–81), and 58 (IQR, 32–74) years for Groups 1, 2, and 3, respectively. The patients in Groups 1 and 2 were older than those in Group 3) (*p* < 0.001). Fewer male patients were seen in Group 3 compared to Group 2 (*p* = 0.024), and black patients were more commonly seen in Group 3 compared to Group 1 (*p* = 0.005).

The patients in Groups 1 and 2 had higher proportions of hypertension (*p* < 0.001 and *p* = 0.004, respectively), diabetes (*p* = 0.021 and *p* = 0.015, respectively), cardiac arrythmias (*p* = 0.002 and *p* < 0.001, respectively), and smoking history (*p* < 0.001 and *p* = 0.016, respectively) than those in Group 3 (Table 2). Chronic kidney disease and coronary heart disease were more commonly seen in Group 2 patients compared to Group 1 patients (*p* = 0.030 and *p* = 0.016, respectively) and Group 3 patients (*p* = 0.012 and *p* < 0.001, respectively). There were no differences in the proportions of patients with active malignancy, history of malignancy, solid organ transplantation, bone marrow transplantation, HIV, previous SARS-CoV-2 infection, or vaccination status across the three groups (Table 2).

**Figure 2:**
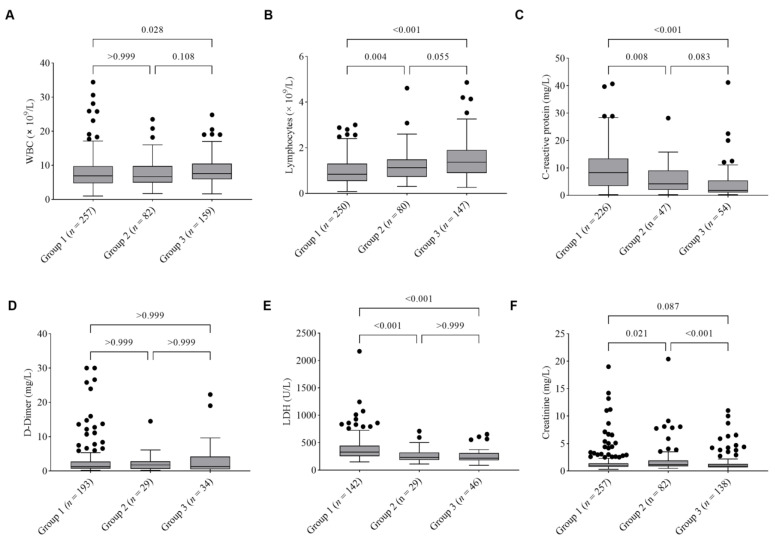
Laboratory characteristics of adults hospitalized for COVID-19 per clinical classifications on admissions, 1–14 January 2022. Box and whisker plots show median values, first quartile and third quartile range of the data and data outliers are denoted by dots. (**A**) Total white blood cell (WBC) count in the blood; (**B**) Absolute lymphocyte count in the blood; (**C**) Serum levels of C-reactive protein; (**D**) Serum levels of D-dimer; (**E**) Serum levels of lactate dehydrogenase (LDH); (**F**) Serum levels of creatinine. *n* is the total number of patients with available data in each group.

Figure 2 shows the medians and interquartile ranges of the key lab findings among the three groups of patients. On admission, the number of WBCs in Group 1 patients was 6.9 (4.7 − 9.7) × 10^9^/L compared to 7.6 (5.9 − 10.5) × 10^9^/L in Group 3 (*p* = 0.028). The number of lymphocytes in Group 1 was 0.8 (0.5 − 1.3) × 10^9^/L compared to 1.1 (0.7 − 1.5) × 10^9^/L in Group 2 (*p* = 0.004) and 1.4 (0.9 − 1.9) × 10^9^/L in Group 3 (*p* < 0.001). The serum levels of CRP were higher in Group 1 compared to that in Group 2 (*p* = 0.008) and Group 3 (*p* < 0.001). The serum levels of LDH were higher in Group 1 compared to that in Group 2 (*p* < 0.001) and Group 3 (*p* < 0.001). The serum levels of creatinine were higher in Group 2 than that in Group 1 (*p* = 0.021) and Group 3 (*p* < 0.001). The serum levels of D-dimer were not different across the three groups of patients.

**Figure 3:**
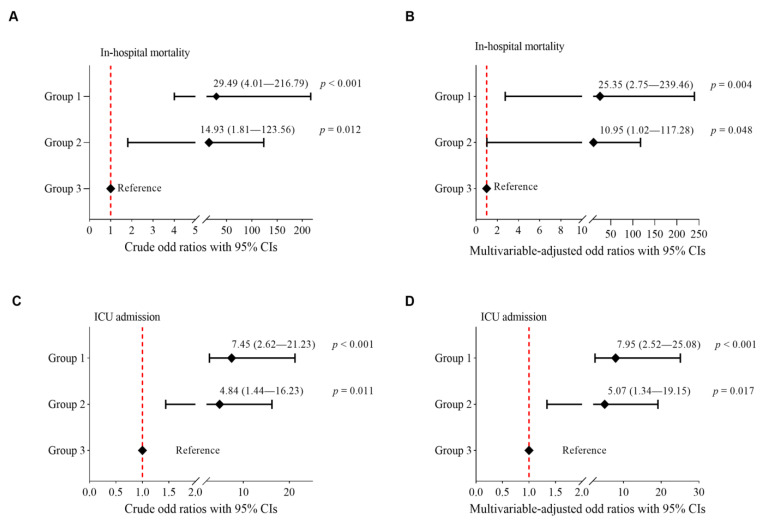
Differences in risk of death and ICU admission by groups. Crude (*left*) and multivariable-adjusted (*right*) odds ratios (ORs) with 95% confidence intervals (CIs) for ICU admission (**C**,**D**) and in-hospital mortality (**A**,**B**) were generated from logistic regression models. The variables included in the multivariable models were age, gender, history of hypertension, diabetes, chronic obstructive pulmonary disease, chronic kidney disease, coronary artery disease, malignancy, transplantation, HIV, vaccination status, and previous SARS-CoV-2 infection.

During hospitalization, a total of 48 out of 500 patients (9.6%) died, and 54 (10.8%) were admitted to the ICU. Patients in Groups 1 and 2 had higher rates of in-hospital death than those in Group 3 (15.6% in Group 1 vs. 0.6% in Group 3, *p* < 0.001; 8.5% in Group 2 vs. 0.6% in Group 3, *p* = 0.002). The in-hospital mortality rates did not differ between Group 1 and Group 2 (15.6% vs. 8.5%, *p* = 0.141). Figure 3 shows the unadjusted and multivariable-adjusted ORs for in-hospital mortality, with Group 3 as a reference. Groups 1 and 2 had significantly higher odds of in-hospital mortality than Group 3, with crude ORs of 29.49 (95% CI, 4.01–216.79; *p* < 0.001) and 14.93 (95% CI, 1.81–123.56; *p* = 0.012), respectively (Figure 3A). After adjusting for age, gender, history of hypertension, diabetes, chronic obstructive pulmonary disease, chronic kidney disease, coronary artery disease, malignancy, transplantation, HIV infection, vaccination status, and previous SARS-CoV-2 infection at admission, Group 1 patients were 26-fold more likely to die during their hospital stay than Group 3 patients (adjusted OR = 25.65, 95% CI, 2.75–239.46; *p* = 0.004), while Group 2 patients were 11-fold more likely to die during their hospital stay than Group 3 patients (adjusted OR = 10.95, 95% CI, 1.02–117.28; *p* = 0.048) (Figure 3B).

Similarly, patients in Groups 1 and 2 had significantly higher rates of admissions to the ICU than those in Group 3 (15.9% in Group 1 vs. 2.5% in Group 3, *p* < 0.001; 10.9% in Group 2 vs. 2.5% in Group 3, *p* = 0.012). The need for ICU admission did not differ between Group 1 and Group 2 (15.9% vs. 10.9%, *p* = 0.371). Figure 3 shows the unadjusted and multivariable-adjusted ORs for ICU admission, with Group 3 as a reference. Patients in Groups 1 and 2 had significantly higher odds of ICU admission than those in Group 3, with unadjusted ORs of 7.45 (95% CI, 2.62–21.23, *p* < 0.001) and 4.84 (95% CI, 1.44–16.23, *p* = 0.012), respectively (Figure 3C). After adjusting for covariates mentioned above, Group 1 patients were 8-fold more likely being admitted to the ICU during their hospital stay than Group 3 patients (adjusted OR = 7.95, 95% CI 2.52–25.08; *p* < 0.001), while Group 2 patients were 5-fold more likely being admitted to the ICU during their hospital stay than Group 3 (adjusted OR = 5.07, 95% CI 1.34–19.15; *p* = 0.017) (Figure 3D).

Table 3 shows the risk of ICU admission and in-hospital death in the study population stratified by age (<65 and ≥65 years) in the different groups. Overall, patients aged ≥ 65 years had a comparable rate of ICU admission (10.2% vs. 11.7%; *p* = 0.585) but a higher rate of in-hospital mortality (12.9% vs. 4.9%; *p* = 0.003) compared to those aged < 65 years, resulting in adjusted ORs of 0.85 (95% CI, 0.49–1.52; *p* = 0.586) and 2.24 (95% CI, 1.10–4.98; *p* = 0.047), respectively. In patients aged < 65 years, those in Groups 1 and 2 had significantly higher rates of admissions to the ICU and in-hospital mortality than those in Group 3 (Table 3), which were consistent with the main analysis shown in Figure 3. In patients aged ≥ 65 years, there were no differences in the risk of ICU admissions among the three groups of patients; however, patients in Group 1 had an increased risk for in-hospital mortality compared to those in Group 3 (adjusted OR = 25.62, 95% CI, 2.32–283.12; *p* = 0.008) (Table 3).

Table 4 shows the effectiveness of COVID-19 vaccines in reducing the risk of severe illness and death compared with unvaccinated patients. Stratified by vaccination status, patients who were fully vaccinated had comparable rates of ICU admission (11.9% vs. 9.6%) and in-hospital mortality (11.2% vs. 7.9%), resulting in adjusted ORs of 1.17 (95% CI, 0.66–2.08; *p* = 0.660) and 1.12 (95% CI, 0.55–2.26; *p* = 0.760), respectively, compared to the unvaccinated patients. The effect of vaccination compared with that of non-vaccination was consistent across almost all subgroups examined (Table 4). The risk of ICU admission and in-hospital mortality did not significantly vary based on the specific dose of vaccination administered (Table 4).

In addition, patients in Groups 1 and 2 had longer hospital LOSs than those in Group 3 (*p* < 0.001 and *p* = 0.002, respectively) (Table 2). The medians and interquartile ranges of hospital LOSs were 6 (IQR, 3–9) days, 5 (IQR, 4–8) days, and 3 (IQR, 2–6) days for patients in Group 1, Group 2, and Group 3, respectively. The ICU LOSs were 10 (IQR, 3.5–16) days, 10 (IQR, 2–18) days, and 2.5 (IQR, 1.3–3.0) days for Group 1, Group 2, and Group 3 patients, respectively. ICU LOSs did not differ among these three groups, despite patients in Groups 1 and 2 showing a trend towards a longer ICU LOS compared to those in Group 3 (Table 2).

Table 5 shows the results of the multivariate regression analysis that indicated the effect of patient-level factors on the odds of prolonged hospital LOSs (>3 days). A longer LOS was associated with those aged ≥65 (OR = 1.72, 95% CI, 1.07–2.77) and those with hypertension (OR = 0.51, 95% CI, 0.31–0.86; *p* = 0.012). Patients who were hospitalized to Group 1 (OR = 2.76; 95% CI, 1.72–4.42; *p* < 0.001) and Group 2 (OR = 2.62; 95% CI, 1.36–5.04; *p* = 0.004) were more likely to be hospitalized for more than 3 days compared to those who were admitted to Group 3.

## 4. Discussion

Initially, studies on patients with the SARS-CoV-2 Omicron variant infection reported decreased severity of disease of COVID-19 as compared to Delta variant infections [3,4,5]. However, these studies did not consider the important differences between patients hospitalized due to COVID-19 infection and those with an incidental COVID-19 diagnosis [4,7]. In this study, we identified that 51.4% of hospitalizations were primary COVID-19 pulmonary cases, 16.4% were COVID-19-associated extrapulmonary problems, and 32.2% were incidental COVID-19 cases. Specifically, patients with COVID-19 infection-causing hospitalizations were older with higher proportions of comorbidities compared to those who were not. In addition, these patients used more health care services and had a higher mortality rate than the incidental COVID-19 cases. Our results provide more insight into understanding the severity of disease and health care utilization associated with the SARS-CoV-2 Omicron variant surge in South Florida.

Being a rapidly evolving RNA virus, the SARS-CoV-2 Omicron variant has a much higher viral infectivity and is more infectious than that of the early wild-type strains and other variants [20]. Initial reports suggest that there is a less severe clinical course with a predominance of milder clinical manifestations such as rhinorrhea, sneezing, sore throat, and headache as compared with the disease caused by previous SARS-CoV-2 variants [3,4,5,21,22]. Clinical characteristics and outcomes of hospitalized patients infected with the Omicron variant showed significantly shorter hospital stays and reduced severity and mortality when compared to the previous COVID-19 waves [4,7]. Our study showed that the clinical characteristics differed significantly in the hospitalized patients with SARS-CoV-2 infections occurring during the period of the Omicron variant circulation. The demographic characteristics of the incidental COVID-19 cases differed significantly from those who were hospitalized due to COVID-19-related symptoms in terms of age and comorbidities. We found that the classifications of hospitalizations correlated with the severity of disease and outcomes, with COVID-19-associated hospitalizations having more adverse outcomes, measured through ICU admissions and in-hospital mortality. Although COVID-19 mRNA vaccines are highly effective in reducing reinfection and preventing the most severe forms of COVID-19 during the Omicron transmission period [6,7,8,23,24], we did not observe an obvious relationship between vaccination and disease severity of COVID-19 in the current study. This may be explained by the fact that the Omicron variant has potent immune escape characteristics, and that the protection offered against infection with the Omicron variant by both vaccination and/or natural infection seems lower than with previous variants [25,26,27]. Waning immunity may also a potential explanation for this finding because the timing of the vaccination was not analyzed in the present study. In our analysis, the “partially vaccinated” individuals were considered as unvaccinated, which may have impacted the results of the study. In addition, the small sample size might not be sufficient to detect differences in disease severity between vaccinated and unvaccinated patients experiencing the Omicron infection, which should be considered when interpreting the results.

Age is a significant risk factor for severe outcomes in COVID-19 cases [28]. Older individuals, particularly those with underlying health conditions, are generally at higher risk of developing severe illness, requiring ICU admission, or experiencing in-hospital death [28,29]. In our study, elderly individuals, typically those aged ≥65 years, were more likely to face a higher mortality rate compared to the younger age group (<65 years). In our subgroup analyses, Groups 1 and 2 patients aged <65 years had a higher risk of both ICU admission and in-hospital death compared to Group 3 patients who were the incidental COVID-19 cases. There was a trend suggesting a higher risk of ICU admission or mortality among patients aged 65 years or older in Groups 1 and 2 compared to Group 3. However, the difference did not reach statistical significance. It is important to note that the small sample size might reduce the ability to detect differences between these subgroups. The numbers of patients, when subdivided into subgroups, might not be sufficient to make definite conclusions for each subgroup; however, the trends for each subgroup were consistent with the overall outcomes of the three groups.

The percentage of hospitalizations classified as incidental COVID-19 varies. Prior to the Omicron variant surge, a report from Los Angeles County Public Health found that 12% of hospitalizations during August–October 2020 were classified as incidental COVID-19 [30]. In a cohort of fully vaccinated patients with breakthrough SARS-CoV-2 infections in Yale New Haven Health System, 46% of hospitalizations were classified as incidental COVID-19 [31]. A study using EHR data from 60 hospitals across four US healthcare systems reported 26% of hospitalizations between March 2020 and August 2021 were classified as incidental COVID-19 [11]. An analysis of all patients with SARS-CoV-2 Omicron variant infections in the Netherlands between 23 December 2021 and 27 February 2022 showed that 31% of hospitalizations were incidental COVID-19 [14]. A population-based surveillance for laboratory-confirmed COVID-19-associated hospitalizations in 99 counties across 14 U.S. states reported that hospitalizations for non-COVID-19-related conditions ranged from 12 to 48% during January–March 2022 [13], and unvaccinated patients were more likely than vaccinated patients to be hospitalized specifically for COVID-19 [32]. In our case series in South Florida during January 2022, 32.2% of hospitalizations were for other reasons, and were “incidentally” found to have COVID-19 infection on routine screening conducted on all hospitalized patients. These data are consistent with the range reported by other sources [13].

Monitoring hospitalizations of COVID-19 has been essential to guide health system planning and public health decision making. People may be hospitalized with, but not because of, SARS-CoV-2 infection, which may result in overcounting COVID-19 hospitalizations with lower rates of in-hospital death. In a study comparing disease severity and clinical outcomes of major SARS-CoV-2 variants, the disease severity associated with the Alpha, Gamma, and Delta variants is comparable, while Omicron infections are significantly less severe [16]. Contrary to these findings, a multicenter prospective cohort study conducted in France between 7 December 2021 and 1 May 2022 showed no significant difference between Delta and Omicron variants for 28-day mortality [17]. In our series, in-hospital mortality was significantly higher in Groups 1 and 2 than in Group 3 patients. Similarly, the need for ICU admission in patients of Groups 1 and 2 was significantly higher than Group 3 patients. The presence of lymphopenia and elevated biomarkers in patients of Groups 1 and 2 is likely a reflection of higher severity of COVID-19 in these groups than those in Group 3 [33,34]. Thus, variations in the proportions of hospitalizations primarily for COVID-19 should be considered when interpreting clinical outcomes for patients with Omicron variant infections. It is important to consider the differences between primary and incidental COVID-19 when studying the outcomes and hospital course of hospitalized COVID-19 patients.

Hospitalizations for primary and incidental COVID-19 cases will have workload implications for healthcare systems, particularly in terms of the volume of patients and the resources required to manage them. Primary COVID-19 is usually more severe and requires more intensive medical care, while incidental COVID-19 patients generally interfere less with routine care. In our series, Group 1 had the highest number of patients that required intensive care, followed by Group 2 and Group 3. There was a trend towards longer ICU stays for Groups 1 and 2 compared to Group 3, despite not reaching statistical difference. The length of hospital stay was significantly shorter for Group 3 compared to Groups 1 and 2. Patients aged ≥65 years had a higher chance of being hospitalized for more than 3 days compared to the age group of <65 years, which is in line with a meta-analysis that showed that older patients were more susceptible to have longer LOSs [35]. These results suggest that the patients in Groups 1 and 2 may require more intensive care and monitoring during their hospitalization than those in Group 3. Counting patients with incidental COVID-19 as primary COVID-19 hospitalizations may result in a skewed reflection of hospital workload and COVID-19 burden. Therefore, it is important to accurately distinguish primary COVID-19 admissions from those who are incidentally found to have COVID-19, in order to better assess the burden of the disease and the resources required to manage it. We recommend healthcare policy makers consider the different groups of hospitalized COVID-19 patients in their decision making.

The findings in this report are subject to several limitations. Our hospitals did not perform sequencing on patient samples to confirm infections with the SARS-CoV-2 Omicron variant, and the diagnosis of COVID-19 was assumed to be caused by the Omicron variant based on the epidemiological data that the Omicron variant of SARS-CoV-2 was the predominant circulating variant in the study period [1]. The classification of COVID-19 hospitalizations was based on a manual review of patients’ records. It is possible that clinicians may have been biased to primary COVID-19 admissions more than incidental COVID-19 cases, which may have impacted our results. The findings presented in this report were derived from data collected from 4 hospitals located in South Florida, which might not be nationally generalizable. Lastly, management of COVID-19 is based on the severity of illness. All of the hospitalized patients in our hospitals received supportive care (supplemental oxygen therapy for SpO2 < 94%) and a variety of therapeutic options, including antiviral medications (e.g., molnupiravir, ritonavir in combination with nirmatrelvir, remdesivir), anti-SARS-CoV-2 monoclonal antibodies (e.g., bamlanivimab/etesevimab, casirivimab/imdevimab, sotrovimab, bebtelovimab), anti-inflammatory drugs (e.g., dexamethasone), and immunomodulators agents, which may have had an impact on patient clinical courses and outcomes.

## 5. Conclusions

This study showed that adult patients hospitalized with the Omicron variant infections exhibited a spectrum of clinical presentations and outcomes. Of the hospitalized adult patients, 32.2% were incidental COVID-19 cases that did not require treatment that was specialized for COVID-19. The patients with primary COVID-19 hospitalizations were older, had more comorbidities as well as higher risks of ICU admission and in-hospital mortality. Some individuals still became infected with SARS-CoV-2 after vaccination. Future studies to investigate risk factors for SARS-CoV-2 reinfections and characterize post-vaccination illness are needed for developing more targeted approaches to reduce the burden of possible future COVID-19 variants on healthcare services.

## Figures and Tables

**Table 1:** Classification criteria of COVID-19 hospitalizations during the Omicron variant surge.

Classification	Definition
Group 1: Primary COVID-19	COVID-19 is the main cause for hospitalization.
	(a) Multilobar COVID-19 pneumonia and hypoxemia.
	(b) COVID-19 respiratory symptoms/less severe pneumonia but on top of other lung disease (COPD/asthma, etc.).
	(c) COVID-19 respiratory symptoms/less severe pneumonia but on top of advanced age/frailty or multiple medical comorbidities.
Group 2: Extrapulmonary manifestations of COVID-19	COVID-19 is associated severe symptoms other than pulmonary presentation.
	(a) COVID-19 with atrial fibrillation or heart failure.
	(b) COVID-19 with acute kidney injury.
	(c) COVID-19 with syncope.
	(d) COVID-19 with severe gastroenteritis.
	(e) COVID-19 and acute cerebrovascular accident.
	(f) COVID-19 with preterm labor/complication of pregnancy.
	(g) Other.
Group 3: Incidental COVID-19	COVID-19 is not the cause for hospitalization, and patient does not have any medical treatment related to COVID-19.
	(a) Psychiatric patients with COVID-19.
	(b) Women in labor or scheduled for caesarean.
	(c) Trauma patients.
	(d) Miscellaneous/Other.

**Table 2:** Characteristics of hospitalized COVID-19 patients by clinical presentations on admission, 1–14 January 2022.

	Group 1	Group 2	Group 3	*p*-Value	*p*-Value	*p*-Value
	(*n* = 257)	(*n* = 82)	(*n* = 161)	Group 1 vs. Group 2	Group 1 vs. Group 3	Group 2 vs. Group 3
Age, years, median (IQR)	73 (61–82)	74 (62–81)	58 (32–74)	0.965	<0.001	<0.001
Male sex	123 (48.2)	44 (53.7)	62 (38.5)	0.361	0.061	0.024
Race				0.654	0.005	0.287
White	176 (68.4)	52 (63.4)	85 (52.8)			
Black	74 (28.8)	28 (34.1)	71 (44.1)			
Other	7 (2.7)	2 (2.4)	5 (3.1)			
Pre-existing health conditions						
Hypertension	186 (72.4)	58 (70.7)	82 (50.9)	0.778	<0.001	0.004
Diabetes	103 (40.1)	37 (45.1)	46 (28.6)	0.804	0.021	0.015
COPD/asthma	48 (18.6)	12 (14.6)	18 (11.2)	0.506	0.536	0.053
Chronic kidney disease	59 (19.4)	29 (35.3)	32 (19.8)	0.030	0.543	0.012
Coronary heart disease	51 (19.8)	27 (32.9)	22 (13.7)	0.016	0.113	<0.001
Cerebrovascular disease	23 (8.9)	9 (10.9)	16 (9.9)	0.664	0.732	0.825
Heart failure	32 (12.5)	14 (17.1)	12 (7.5)	0.353	0.139	0.028
Cardiac arrythmias	47 (18.2)	23 (28)	12 (7.5)	0.062	0.002	<0.001
Active malignancy	14 (5.4)	8 (9.8)	9 (5.6)	0.197	>0.999	0.287
History of malignancy	30 (11.7)	9 (10.9)	13 (8.1)	>0.999	0.252	0.483
Solid organ transplantation	4 (1.6)	4 (4.9)	2 (1.2)	0.100	>0.999	0.183
Bone marrow transplantation	1 (0.4)	2 (2.4)	0 (0)	0.146	>0.999	0.112
HIV infection	2 (0.8)	1 (1.2)	3 (1.9)	0.565	0.377	>0.999
Smoking	93 (36.9)	27 (32.9)	30 (18.6)	0.691	<0.001	0.016
Obesity	111/254 (43.7)	30/81 (37.0)	38/130 (29.2)	0.304	0.007	0.289
Previous COVID-19 infection	13/252 (5.2)	4/77 (5.2)	15/149 (10.1)	>0.999	0.070	0.312
COVID-19 vaccination status				0.306	0.192	0.056
Fully vaccinated	137 (53.3)	48 (58.5)	75 (46.5)			
Unvaccinated	120 (46.7)	34 (41.5)	86 (53.5)			
Hospital LOS, days, median (IQR)	6 (3–9)	5 (4–8)	3 (2–6)	>0.999	<0.001	0.002
ICU LOS, days, median (IQR)	10 (3.5–16)	10 (2–18)	2.5 (1.3–3.0)	>0.999	0.055	0.189

Data given as median (interquartile range, IQR), *n* (%), or n/N (%), where N is the total number of patients with available data. COPD, chronic obstructive pulmonary disease; HIV, human immunodeficiency virus; COVID-19, coronavirus disease 2019; LOS, length of stay; ICU, intensive care unit.

**Table 3:** Risk of ICU admission and in-hospital death in hospitalized patients with Omicron variant infections, 1–14 January 2022, stratified by age.

	Age Status	Number of Patients, *n* (%)	Admission to ICU		*p*-Value	In-Hospital Death		*p*-Value
			Yes, *n* (%)	Adjusted OR (95% CI)		Yes, *n* (%)	Adjusted OR (95% CI)	
Overall	<65 years	205 (41)	24 (11.7)	Reference		10 (4.9)	Reference	
	≥65 years	295 (59)	30 (10.2)	0.85 (0.49–1.52)	0.586	38 (12.9)	2.24 (1.10–4.98)	0.047
Subgroup analyses by age group								
Age < 65 years	Group 1	83 (40.5)	19 (22.9)	39.78 (1.13–308.17)	<0.001	8 (9.6)	Infinity (2.18–infinity)	0.002
	Group 2	22 (10.7)	3 (13.6)	12.02 (1.07–135.08)	0.044	2 (9.1)	Infinity (2.16–infinity)	0.034
	Group 3	100 (48.8)	2 (2.0)	Reference		0 (0.0)	Reference	
Age ≥ 65 years	Group 1	179 (60.7)	22 (12.3)	4.91 (0.98–24.48)	0.052	32 (17.9)	25.62 (2.32–283.12)	0.008
	Group 2	61 (20.7)	6 (9.8)	3.60 (0.61–21.19)	0.156	5 (8.2)	7.67 (0.57–103.31)	0.124
	Group 3	55 (18.6)	2 (3.6)	Reference		1 (1.8)	Reference	

OR, odds ratio; CI, confidence interval; ICU, intensive care unit; BMI, body mass index.

**Table 4:** Impact of vaccination on severity outcomes in hospitalized patients with Omicron variant infections, 1–14 January 2022.

	Vaccination Status	Number of Patients, *n* (%)	Admission to ICU		*p*-Value	In-Hospital Death		*p*-Value
			Yes, *n* (%)	Adjusted OR (95% CI)		Yes, *n* (%)	Adjusted OR (95% CI)	
Overall	Unvaccinated	240 (48)	23 (9.6)	Reference		19 (7.9)	Reference	
	Fully vaccinated	260 (52)	31 (11.9)	1.17 (0.66–2.08)	0.600	29 (11.2)	1.12 (0.55–2.26)	0.760
Subgroup analyses by number of doses of COVID-19 vaccine received								
1 dose	Unvaccinated	187 (77.9)	15 (8.0)	Reference		12 (6.4)	Reference	
	One dose	53 (22.1)	8 (15.1)	1.15 (0.41–3.28)	0.790	7 (13.2)	2.13 (0.67–6.77)	0.197
2 doses	Unvaccinated	187 (58.4)	15 (8.0)	Reference		12 (6.4)	Reference	
	Two doses	190 (41.6)	19 (10.0)	1.39 (0.68–2.87)	0.366	16 (8.4)	1.67 (0.77–3.63)	0.194
3 doses	Unvaccinated	187 (72.8)	15 (8.0)	Reference		12 (6.4)	Reference	
	Three doses	70 (27.2)	4 (5.7)	0.45 (0.14–1.53)	0.205	4 (5.7)	0.88 (0.28–2.73)	0.833
Comparison analyses by number of doses of COVID-19 vaccine received								
Number of doses of COVID-19 vaccine	One dose	53 (16.9)	8 (15.1)	Reference		7 (13.2)	Reference	
	Two doses	190 (60.7)	19 (10.0)	0.89 (0.37–2.16)	0.893	16 (8.4)	0.54 (0.25–2.91)	0.788
	Three doses	70 (22.4)	4 (5.7)	0.37 (0.09–1.43)	0.150	4 (5.7)	0.38 (0.07–1.94)	0.245

OR, odds ratio; CI, confidence interval; ICU, intensive care unit; BMI, body mass index.

**Table 5:** Univariate and multivariate regression analysis of factors affecting hospital length of stay.

	Univariate Analysis		Multivariate Analysis	
Variables	OR (95% CI)	*p*-Value	OR (95% CI)	*p*-Value
Age (years)				
≥65 years	2.21 (1.50–3.26)	<0.001	1.72 (1.07–2.77)	0.025
<65 years	1.00		1.00	
Sex				
Male	1.04 (0.71–1.53)	0.834	-	-
Female	1.00			
Race				
Black	0.82 (0.55–1.23)	0.335	-	-
Other	1.21 (0.12–11.79)	0.869	-	-
White	1.00			
BMI (kg/m^2^)				
>30	1.18 (0.78–1.79)	0.433	-	-
≤30	1.00			
Diabetes	1.32 (0.89–1.98)	0.166	-	-
Hypertension	1.17 (0.79–1.74)	0.444	0.51 (0.31–0.86)	0.012
COPD/asthma	1.31 (0.64–2.67)	0.456	-	-
Chronic kidney disease	1.09 (0.69–1.72)	0.696	-	-
Coronary heart disease	1.51 (0.91–2.51)	0.108	-	-
Cerebrovascular disease	0.97 (0.51–1.84)	0.918	-	-
Chronic liver disease	1.68 (0.55–5.16)	0.362	-	-
Heart failure	1.02 (0.58–1.82)	0.935	-	-
Cardiac arrythmias	1.56 (0.90–2.72)	0.112	-	-
Active malignancy	1.08 (0.49–2.41)	0.845	-	-
History of malignancy	1.73 (0.86–3.46)	0.122	-	-
History of solid organ transplantation	4.97 (0.64–38.89)	0.126	-	-
History of bone marrow transplantation	0.88 (0.08–9.79)	0.918	-	-
HIV infection	0.44 (0.09–2.18)	0.313	-	-
Previous COVID-19 infection	0.92 (0.42–1.98)	0.813	-	-
COVID-19 vaccination	1.37 (0.22–1.44)	0.118	-	-
Smoking	0.78 (0.43–1.87)	0.781	-	-
Type of COVID-19 hospitalizations				
Group 1	3.63 (2.36–5.57)	<0.001	2.76 (1.72–4.42)	<0.001
Group 2	3.51 (1.92–6.45)	<0.001	2.62 (1.36–5.04)	0.004
Group 3	1.00		1.00	

OR, odds ratio; CI, confidence interval; BMI, body mass index; COPD, chronic obstructive pulmonary disease; HIV, human immunodeficiency virus.

## Data Availability

The dataset analyzed in the current study is not publicly available due to regulations for protecting patient privacy and confidentiality. The anonymized data are available from the authors upon reasonable request, and after approval from the Memorial Healthcare System institutional review board.
